# Novel Antihypertensive Medications to Target the Renin-Angiotensin System: Mechanisms and Research

**DOI:** 10.31083/RCM27963

**Published:** 2025-04-21

**Authors:** Zhe Jiang, Changlin Zhai, Guanmin Tang

**Affiliations:** ^1^Department of Cardiology, Jiaxing University Master Degree Cultivation Base, Zhejiang Chinese Medical University, 310053 Hangzhou, Zhejiang, China; ^2^Department of Cardiology, The Affiliated Hospital of Jiaxing University, 314001 Jiaxing, Zhejiang, China

**Keywords:** hypertension, anti-hypertensive agents, renin-angiotensin system

## Abstract

An estimated 1.28 billion individuals in the global population suffer from hypertension. Importantly, uncontrolled hypertension is strongly linked to various cardiovascular and cerebrovascular diseases. The role of the renin-angiotensin system (RAS) is widely acknowledged in the development and progression of hypertension. This system comprises angiotensinogen, the renin/(pro)renin/(pro)renin receptor (PRR) axis, the renin/angiotensin-converting enzyme/angiotensin (Ang) II/Ang II type I receptor (AT1R) axis, the renin/angiotensin-converting enzyme (ACE) 2/Ang (1-7)/Mas receptor (MasR) axis, the alamandine/Mas-related G protein-coupled D (MrgD) receptor axis, and the renin/ACE/Ang II/Ang II type II receptor (AT2R) axis. Additionally, brain Ang III plays a vital role in regulating central blood pressure. The current overview presents the latest research findings on the mechanisms through which novel anti-hypertensive medications target the RAS. These include zilebesiran (targeting angiotensinogen), PRO20 (targeting the renin/(pro)renin/PRR axis), sacubitril/valsartan (targeting the renin/ACE/Ang II/AT1R axis), GSK2586881, Ang (1-7) and AVE0991 (targeting the renin/ACE2/Ang (1-7)/MasR axis), alamandine (targeting the alamandine/MrgD receptor axis), C21 and β-Pro7-Ang III (targeting the renin/ACE/Ang II/AT2R axis), EC33, and firibastat and NI956 (targeting brain Ang III).

## 1. Introduction

**Fig. 1.**
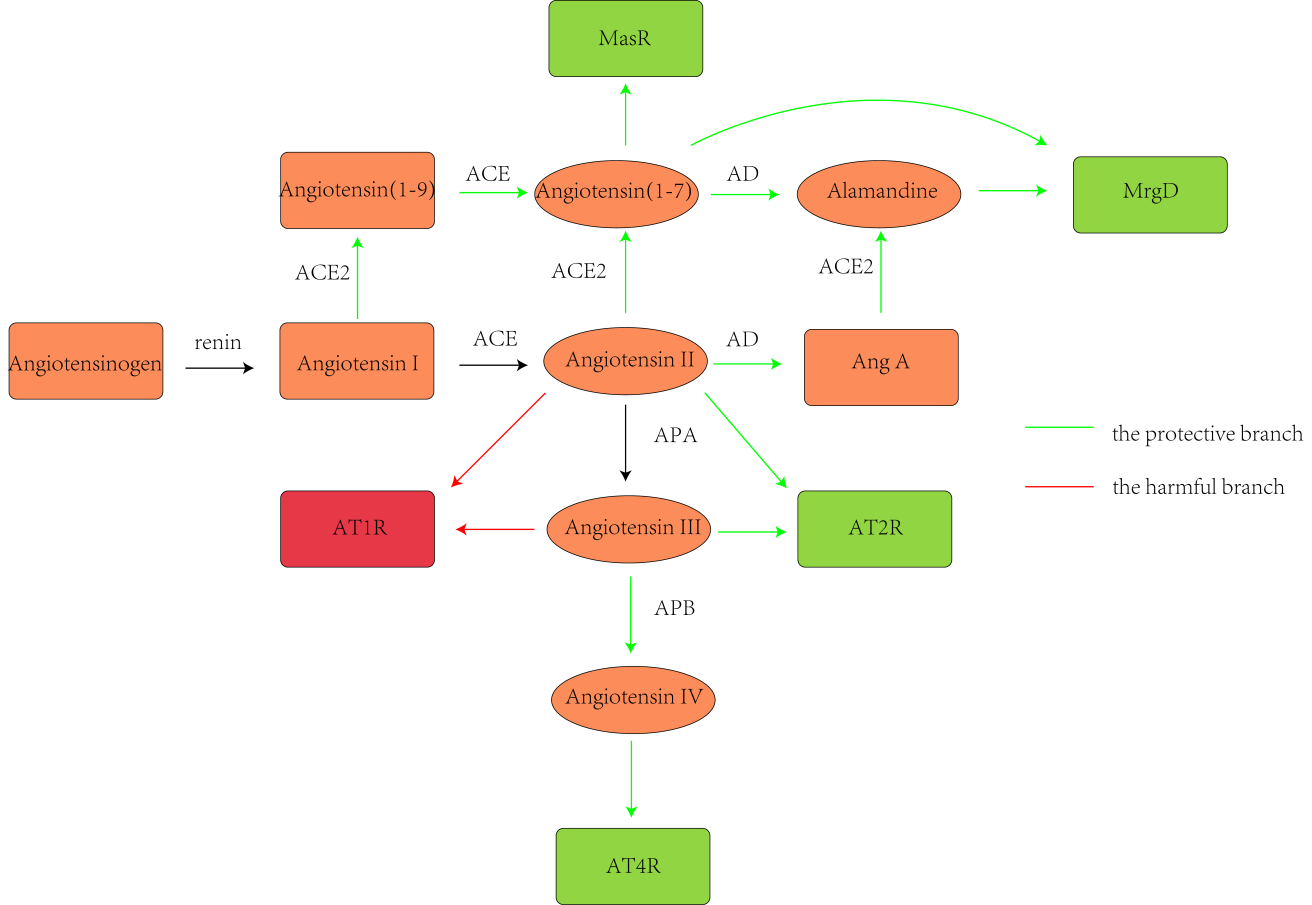
**The renin-angiotensin system (RAS) pathways**. Renin cleaves angiotensinogen to form angiotensin (Ang) I, which is further proteolyzed by angiotensin-converting enzyme (ACE) to produce Ang II. In the harmful branch of the RAS, Ang II can directly stimulate Ang II type I receptor (AT1R), leading to increased blood pressure (BP) through mechanisms such as blood vessel constriction, increased release of aldosterone and arginine-vasopressin, reduced baroreflex responsiveness, lower nitric oxide (NO) production, and decreased sodium excretion. Additionally, AT1R activation can occur through Ang III, which is generated when aminopeptidase A (APA) acts on Ang II. Conversely, the protective branch of the RAS involves the stimulation of Mas receptor (MasR), Mas-related G protein-coupled D (MrgD), Ang II type II receptor (AT2R), and Ang II type IV receptor (AT4R), primarily via NO, thereby inducing vasodilation and natriuresis to lower BP. MasR is mainly activated by Ang (1-7), formed through Ang II cleavage by ACE2, or ACE action on Ang (1-9). Ang (1-9) is formed predominantly through the cleavage of Ang I by ACE2. Moreover, Ang (1-7) can be cleaved by aspartate decarboxylase (AD) to produce alamandine, thereby activating MrgD and reducing BP. Alamandine can also be generated from Ang A by ACE2, following synthesis from Ang II by AD. Furthermore, AT2R can be activated by both Ang II and Ang III, with Ang III cleaved by aminopeptidase B (APB) to produce Ang IV, thus activating AT4R. Created with Adobe Illustrator 2023.

The global population of individuals aged 30 to 79 years who suffer from hypertension is estimated to be around 1.28 billion. Approximately 46% of individuals overlook this condition due to the absence of noticeable symptoms [1]. It is important to note that uncontrolled hypertension is strongly linked to various cardiovascular and cerebrovascular diseases, including myocardial infarction, cardiac insufficiency, cerebral vascular accident, terminal renal disease, and premature death. To mitigate these risks, it is therefore to maintain blood pressure (BP) within the targeted range. Numerous studies have demonstrated a crucial role for the renin-angiotensin system (RAS) in BP regulation [2]. The RAS is the most important and extensively studied hormonal system involved in the development and progression of hypertension [3]. It consists of angiotensinogen (AGT), the renin/(pro)renin/(pro)renin receptor (PRR) axis, the renin/angiotensin-converting enzyme (ACE)/angiotensin (Ang) II/Ang II type I receptor (AT1R) axis, the renin/ACE2/Ang (1-7)/Mas receptor (MasR) axis, the Alamandine/Mas-related G protein-coupled D (MrgD) receptor axis, and the renin/ACE/Ang II/Ang II type II receptor (AT2R) axis (Fig. 1). Brain tissue contains all components of the systemic RAS [4], including AGT, enzymes (renin, ACE, ACE2, aminopeptidase A (APA), aminopeptidase B (APB)), peptides (Ang I, Ang II, Ang III, Ang IV, Ang (1-7)), and angiotensin receptors (AT1R, AT2R, and MasR). In particular, brain Ang III plays a vital role in central BP regulation. The RAS exerts its regulatory role by acting on the above pathways via both systemic and local mechanisms. With regard to the systemic mechanism, increasing evidence shows the renin/ACE2/Ang (1-7)/MasR axis has anti-inflammatory and anti-fibrotic effects. Moreover, the renin/ACE/Ang II/AT1R axis exacerbates inflammation and fibrosis. Several studies have reported imbalances in the ACE-ACE2 ratio in heart and kidney fibrosis, and reduced ACE2 expression and upregulation of Ang II in pulmonary inflammation [5, 6]. With regard to the local mechanism, the RAS has been linked to hypertension in the cardiovascular system, dyslipidemia in adipocyte metabolism, memory and cognitive functions in the central nervous system, glomerulosclerosis and tubulointerstitial fibrosis in the kidney, and mucosal barrier integrity in the gastrointestinal tract [7]. Several anti-hypertensive drugs that target the RAS are in use, including ACE-inhibitors (ACEi) and AT1R blockers (ARB). However, the most recent direct renin inhibitor, aliskiren, was approved by the Food and Drug Administration (FDA) more than 15 years ago. Recent research has led to the development of a growing number of new anti-hypertensive medications that target the RAS. This overview presents the latest research findings and mechanisms for novel anti-hypertensive medications that target the RAS, including zilebesiran, PRO20, sacubitril/valsartan, GSK2586881, Ang (1-7), AVE0991, Alamandine, C21, β-Pro7-Ang III, EC33, firibastat, and NI956.

## 2. Focus on AGT

AGT is primarily synthesized by the liver and serves as the sole precursor for all angiotensin peptides [8]. Therefore, inhibiting the production of AGT in the liver could be a promising treatment for hypertension by decreasing or potentially eliminating the production of the powerful vasoconstrictor Ang II.

### Zilebesiran

**Fig. 2.**
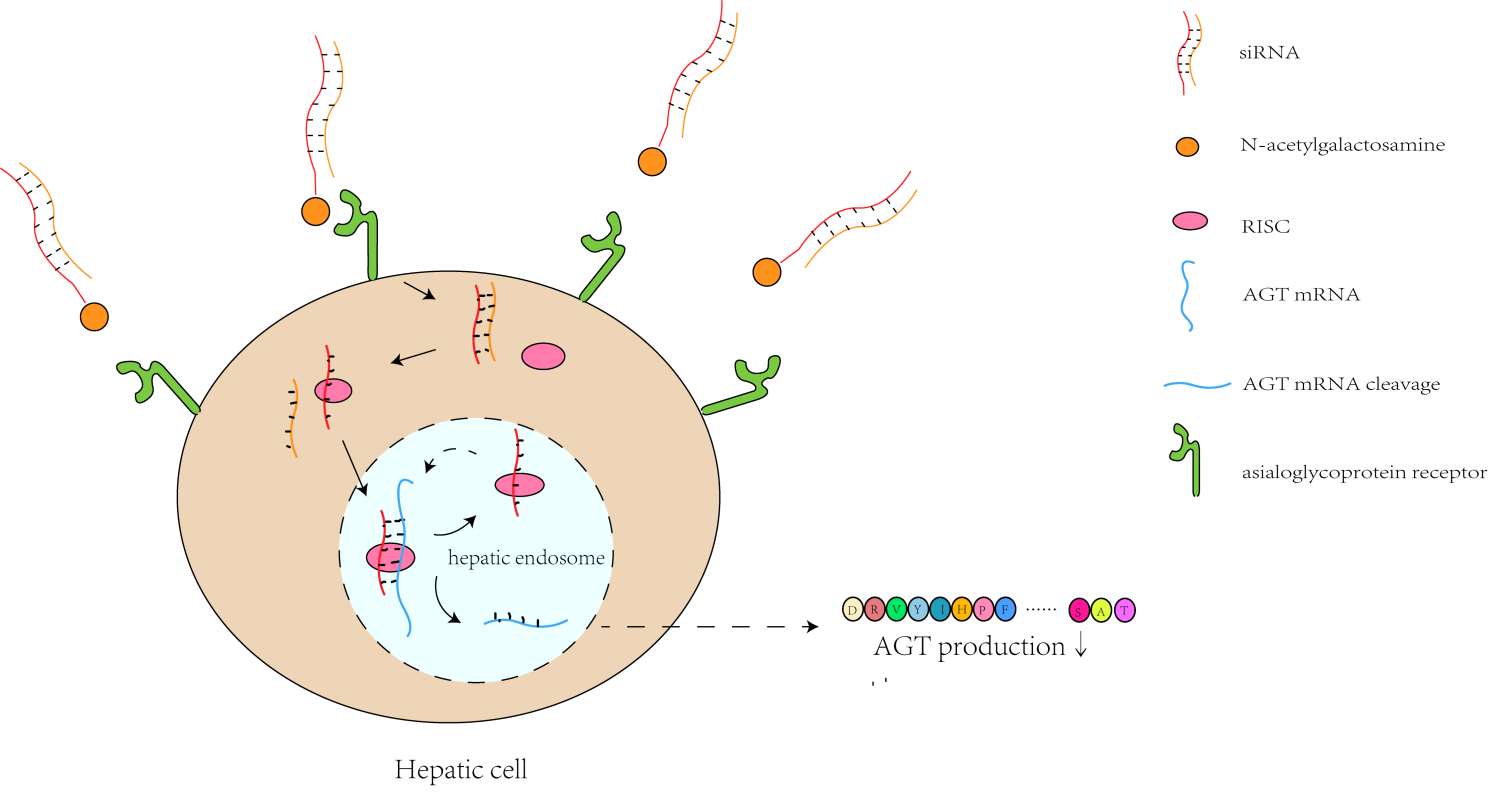
**The mechanism of zilebesiran in hepatic cells**. After binding to asialoglycoprotein receptors on the cell surface, zilebesiran enters the cells through endocytosis. It then attaches to RNA-induced silencing complex (RISC), and the small interfering RNA (siRNA) separates into passenger and guide strands. The passenger strand is broken down, while the guide strand enables activated RISC to bind the angiotensinogen (AGT) messenger RNA (mRNA). Upon binding, the AGT mRNA is degraded, thus reducing the production of AGT. “↓” in the Fig. 2 means the reduction of AGT production. Created with Adobe Illustrator 2023.

Recently, several new drugs based on small interfering RNA (siRNA) have been developed. These include inclisiran, a novel lipid-reducing medication. An animal study found that AGT siRNA significantly reduced BP in spontaneously hypertensive rats (SHRs) and normotensive rats, with no liver or kidney injury [9]. It was reported that AGT siRNA reduced circulating AGT by almost 98% and was as effective in lowering BP as single RAS blockade, without significantly lowering plasma or renal Ang II levels. Zilebesiran is the first siRNA aimed at inhibiting AGT production in the liver, and can effectively reduce the production of Ang I and Ang II. The siRNA in zilebesiran is conjugated to N-acetylgalactosamine, an amino sugar that binds to asialoglycoprotein receptors on the surface of hepatocytes. This ensures drug delivery to the liver rather than to other organs such as the kidneys, brain, adrenal glands, etc., [10]. Zilebesiran enters the cell via endocytosis and subsequently attaches to the RNA-induced silencing complex (RISC) located in the cytoplasm. Following integration, the siRNA separates into passenger and guide strands, and the former is then degraded. The guide strand pairs with AGT messenger RNA (mRNA), thus enabling it to combine with activated RISC. After combining, the AGT mRNA is degraded, effectively preventing the translation of AGT. The peak effect on circulating AGT is reached by week 3, and on BP by week 8 due to the time required for sufficient cleavage of AGT mRNA and hence the depletion of AGT [11]. When attached to the RISC, the siRNA is shielded from nuclease degradation, allowing it to be recycled and leading to multiple degradation cycles of AGT mRNA. This results in a sustained anti-hypertensive effect for 6 months [12, 13] (Fig. 2). Desai *et al*. [13] conducted a double-blind, randomized, placebo-controlled phase 1 clinical trial to evaluate the safety, pharmacokinetics, and pharmacodynamics of zilebesiran in hypertensive patients. A total of 84 participants were randomly assigned to receive either a single increasing dose of zilebesiran (10, 25, 50, 100, 200, 400, or 800 mg), or a placebo. Following the administration of single, subcutaneous doses of zilebesiran, sustained decreases in serum AGT levels and BP were observed in a dose-dependent manner for up to 6 months. Reductions in systolic blood pressure (SBP; >10 mmHg) and diastolic blood pressure (DBP; >5 mmHg) were observed by week 8 following a single administration of zilebesiran at a dosage of 200 mg or higher. In patients administered 800 mg of zilebesiran, the average SBP and DBP decreased by –22.5 ± 5.1 mmHg and –10.8 ± 2.7 mmHg, respectively, at week 24 [13]. The KARDIA-1 phase 2 study included 394 patients with mild to moderate hypertension. It also found the BP-lowering effects of zilebesiran were still present 6 months after receiving a single dose, especially 300 mg and 600 mg. Patients who received doses of 300 mg or higher showed a stronger ability to lower AGT levels (by >90%) at 6 months compared to the 800 mg dose in the phase 1 trial, suggesting that lower doses may be effective [14]. Regarding the safety of zilebesiran, only minor injection site reactions were reported in the phase 1 trial, with no hypotension, hyperkalemia, or deterioration of kidney function [13]. During the phase 2 trial, patients treated with zilebesiran experienced a higher frequency of injection site reactions and hyperkalemia compared to those who received placebo, but the majority of adverse reactions were mild [14]. In view of its persistent anti-hypertensive effect at 6 months and its acceptable tolerability, zilebesiran could effectively improve adherence and reduce therapeutic inertia, with no significant adverse effects compared to traditional anti-hypertensive medications. Zilebesiran is still in the preliminary stages of the FDA approval process, and its efficacy and safety relative to current standard anti-hypertensive therapy must still be determined.

## 3. Focus on the Renin/(Pro)renin/PRR Axis

The renin/(pro)renin/PRR axis is an upstream component of the RAS and plays a vital role in its activation. Renin is produced by cathepsin B-mediated removal of the N-terminal pro-segment from its inactive precursor prorenin. Renin plays an important role in the regulation of BP via its production of Ang II [15]. The PRR protein consists of 350 amino acids and contains a single transmembrane domain. PRR is expressed at high levels in the heart and brain, but at low levels in the kidney and liver [16]. PRR-bound renin and prorenin exhibit high catalytic activity and stimulate the production of Ang II [17]. Accumulating evidence shows that activation of PRR is closely associated with hypertension. The inhibition of PRR could therefore potentially exert an anti-hypertensive effect.

### PRO20

**Fig. 3.**
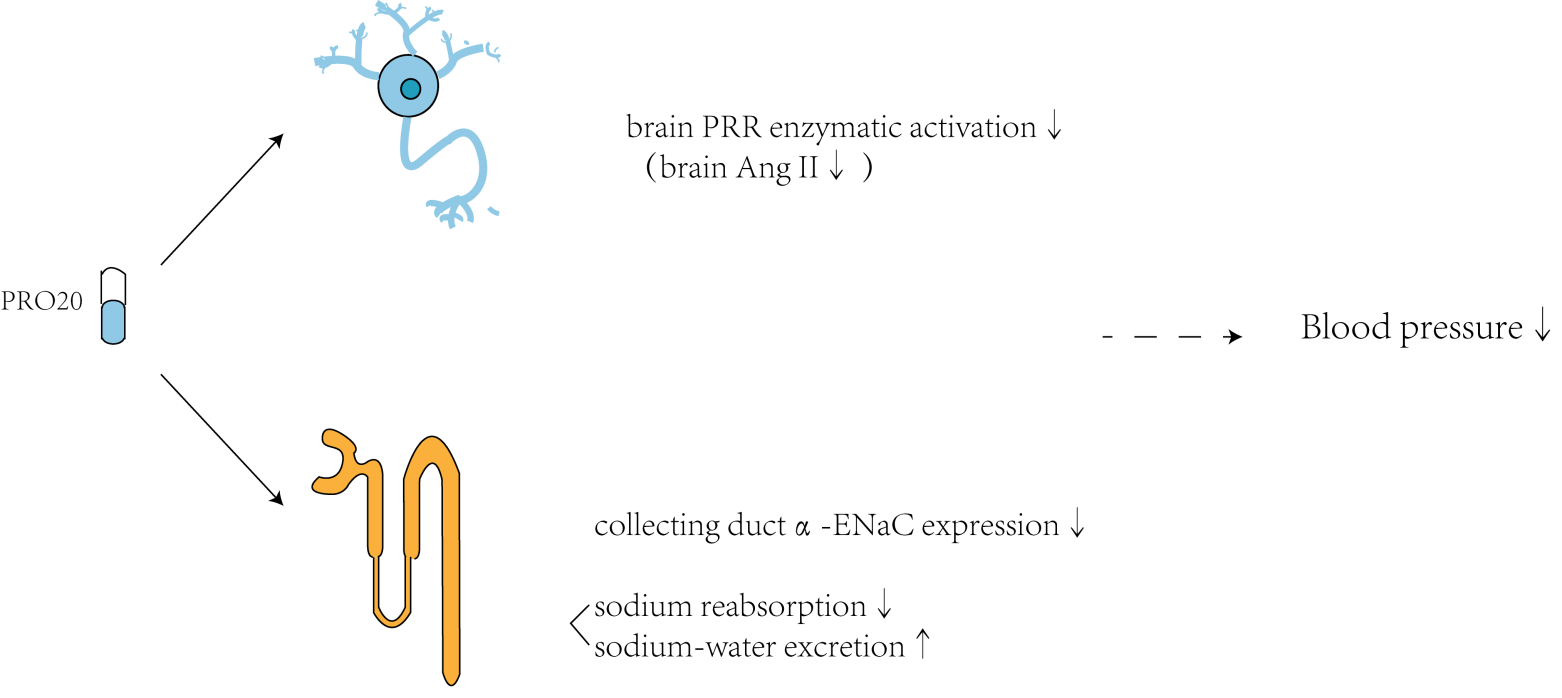
**Hypotensive mechanisms involving PRO20 targeting of the renin/(pro)renin receptor (PRR) axis**. On one hand, PRO20 can prevent activation of the PRR enzyme in the brain by blocking calcium influx in neurons and decreasing the levels of Ang II in the brain stem, cortex, and hypothalamus, thereby exerting an anti-hypertensive effect. On the other hand, PRO20 can decrease the expression of α-epithelial sodium channel (α-ENaC) in the collecting duct to reduce sodium reabsorption and enhance sodium-water excretion. It does this by inhibiting the activation of renal PRR and the intrarenal RAS. “Brain PRR enzymatic activation↓” means the decrease of brain PRR enzymatic activation; “brain Ang II↓” means the reduction of the brain Ang II production; “collecting duct α-ENaC expression↓” means the decrease of collecting duct α-ENaC expression; “sodium reabsorption↓” means the reduction of sodium reabsorption; “sodium-water excretion↑” means the increase of sodium-water excretion; “blood pressure↓” means the decrease of blood pressure. Created with Adobe Illustrator 2023.

PRO20 consists of the initial 20 amino acid residues of the (pro)renin pro-segment. PRO20 can effectively inhibit PRR by specifically blocking the interaction between renin/prorenin and PRR, with emerging evidence that it could potentially attenuate hypertension. It was reported that injecting PRO20 directly into the brain ventricles of prorenin-induced hypertensive C57Bl6/J mice blocked calcium influx into neurons, thus preventing activation of the PRR enzyme and reducing hypertension. Acute or chronic intra-cerebroventricular (ICV) administration of PRO20 was found to lower BP in both deoxycorticosterone acetate-salt (DOCA)-salt and human renin–angiotensinogen double-transgenic hypertensive mice. Additionally, PRO20 decreased the level of Ang II in the brain stem, cortex, and hypothalamus. In addition to its effect on PRR in the brain, PRO20 may also exert its anti-hypertensive effect by targeting renal PRR (Fig. 3). Intramedullary and intravenous administration of PRO20 were compared in Ang II-induced high hypertensive mice. Intramedullary PRO20 infusion (IM PRO20) significantly reduced Ang II-induced hypertension with no apparent toxicity, while showing a more efficient BP-lowering effect than intravenous PRO20 infusion [18]. Fu *et al*. [19] demonstrated that subcutaneous administration of PRO20 in late pregnant mice effectively attenuated the upregulation of pregnancy-induced α-epithelial sodium channel (α-ENaC) and sodium-water retention in the renal collecting duct, while reducing intrarenal RAS markers. Hence, PRO20 can decrease the expression of α-ENaC to reduce sodium reabsorption and enhance sodium-water excretion by preventing the activation of renal PRR and intrarenal RAS. Earlier studies demonstrated that PRO20 exerted a strong anti-hypertensive effect by targeting the brain and renal PRR. However, in more recent studies PRO20 was delivered locally, thus guaranteeing a sufficient local concentration in the brain or kidney. The high molecular weight of the PRO20 peptide limits its ability to traverse the blood-brain barrier (BBB) and inhibit the PRR in the brain. Another study showed that intravenous administration of PRO20 (IV PRO20) was significantly less effective at reducing mean arterial pressure (MAP) than IM PRO20 [18]. In addition to its anti-hypertensive effect, PRO20 also exerts reno-protective effects in the 5/6 nephrectomy rat model by preventing activation of renal Wnt/β-catenin signaling. This pathway is known to play a key role in the pathogenesis of chronic kidney disease (CKD) [3]. Compared to traditional anti-hypertensive medicine, PRO20 could therefore exert additional reno-protective effects which may benefit hypertensive patients with accompanying CKD. To date, few pharmacokinetic studies or clinical trials have been conducted on PRO20, and further in-depth studies of this agent are warranted.

## 4. Focus on the Renin/ACE/Ang II/AT1R Axis

It is well established that ARB exerts its anti-hypertensive effects by inhibiting AT1R. Neprilysin is a membrane-bound endopeptidase that hydrolyzes natriuretic peptides including atrial natriuretic peptide and brain natriuretic peptides, causing vasodilation and favoring the excretion of salt and water to regulate BP [7]. Neprilysin inhibitor (NEPi) could therefore be a potential therapeutic option by increasing endogenous peptide levels. However, NEPi alone does not cause a clinically meaningful reduction in BP, possibly due to neprilysin-dependent breakdown of polypeptide vasoconstrictors such as Ang II. To prevent this adverse effect, NEPi can be used in combination with ARB to avoid the undesirable effect of increased Ang II. A novel medication for treating hypertension and composed of an ARB and a NEPi was therefore developed: angiotensin receptor neprilysin inhibitor (ARNI). In patients with hypertension, administration of ARNI resulted in a greater reduction in BP compared to valsartan alone. This agent was approved by the FDA for hypertension treatment in 2015.

### Sacubitril/Valsartan

The first ARNI, sacubitril/valsartan, was developed by the Novartis pharmaceuticals corporation. It is metabolized separately into valsartan and sacubitril by the body. Sacubitril is further metabolized by esterase into the active NEPi LBQ657. Approximately 37 to 48% of sacubitril/valsartan is excreted in urine and 52 to 68% in feces, mostly as LBQ657. Feces contain about 86% of the metabolites. The mean elimination half-lives of valsartan, sacubitril, and LBQ657 were approximately 9.9 h, 1.4 h, and 11.5 h, respectively [20]. A meta-analysis of 9 randomized controlled trials involving 6765 subjects demonstrated that sacubitril/valsartan significantly lowered SBP (weighted mean difference (WMD): –4.11 mmHg), DBP (WMD: –1.79 mmHg), mean 24 h SBP (WMD: –3.24 mmHg) and mean 24 h DBP (WMD: –1.25 mmHg) compared to ARB (including valsartan and olmesartan). Moreover, no differences were observed in the frequency of adverse events between the sacubitril/valsartan group and the ARB/placebo group [21]. A recent randomized, multicenter, open-label, non-inferiority trial (PARASOL study) evaluated the efficacy and safety of sacubitril/valsartan versus amlodipine in Japanese patients with hypertension. The average 24 h SBP reduction in sacubitril/valsartan from baseline to week 8 was found to be non-inferior to that of amlodipine (between-treatment difference: –0.62 mmHg) [22]. Sacubitril/valsartan also exerted an anti-hypertensive effect in patients with both hypertension and CKD, significantly reducing SBP by 20.5 mmHg and the urinary albumin/creatinine ratio by 15.1% (estimated glomerular filtration rate (eGFR) ≥15 and ≤60 mL⋅min^-1^⋅1.73 m^-2^). Moreover, no clinically meaningful changes were observed in creatinine, potassium, blood urea nitrogen and eGFR [23]. Another recent clinical trial found that sacubitril/valsartan significantly reduced proteinuria and SBP in patients with stage 4–5 CKD, without significantly changing eGFR, serum potassium, and serum uric acid [24]. In the PARADIGM-HF clinical trial, sacubitril/valsartan displayed superior cardio-protective effects compared to ACEi monotherapy [25]. With regard to the side effects of sacubitril/valsartan other than angioedema, the long-term inhibition of neprilysin may adversely impact conditions such as bronchial reactivity, inflammation and cancer, and increase susceptibility to polyneuropathy [26]. Overall, sacubitril/valsartan could exert a stronger BP-lowering effect compared to traditional anti-hypertensive medications because of its dual anti-hypertensive mechanism. Furthermore, sacubitril/valsartan exhibits significant cardio-protective and reno-protective effects.

## 5. Focus on the Renin/ACE2/Ang (1-7)/MasR Axis

The renin/ACE2/Ang (1-7)/MasR axis, and in particular ACE2 and Ang (1-7), are promising targets for therapy. ACE2 shares 40% similarity with ACE and is predominantly located in the vascular endothelium of the kidney, heart, brain, and testis [27, 28, 29, 30, 31]. Despite its limited plasma activity, ACE2 can convert Ang II into Ang (1-7) by promoting translation [32]. ACE2 can also cleave other peptide substrates, such as Ang I to Ang (1-9), and Ang-A to alamandine [33]. After binding to G-protein-coupled MasR, Ang (1-7) exhibits anti-hypertensive, anti-apoptotic, anti-inflammatory, and anti-thrombotic effects on the cardiovascular system. ACE2 is closely associated with hypertension, and its mRNA and protein levels are markedly reduced in SHRs compared with normative rats [34]. Another preclinical trial showed that ACE2 knockout mice demonstrated a lower basal BP than wildtype mice. This may be explained by endothelial dysfunction, with reduced nitric oxide (NO) and increased generation of reactive oxygen species. The expression of ACE2 in the brain may also be involved in its cardiovascular actions, since ACE2 deletion increases oxidative stress in the brain and activates the sympathetic nervous system [35, 36]. Several novel anti-hypertensive medications targeting these mechanisms have been developed, including GSK2586881, Ang (1-7), and AVE0991.

### 5.1 GSK2586881

GSK2586881 is a soluble recombinant human variant of the naturally existing ACE2 enzyme. Intravenous infusion of GSK2586881 was found to enhance ACE2 enzymatic function [37]. Following subcutaneous injection in mice, GSK2586881 is detected in the plasma, indicating that it can be absorbed either by lymphatic vessels or directly across capillary walls. In healthy human subjects, exposure to GSK2586881 is dose-dependent, with a slightly higher volume of distribution than plasma, and elimination with a terminal half-life of about 10 h [37]. Increased GSK2586881 levels prolong the lowering of Ang II levels for an extended period, while also maintaining elevated Ang (1-7) levels for at least 24 h. Systemic exposure to GSK2586881 appears to increase almost in proportion to the dosage, ranging from 0.1 to 0.8 mg/kg [38]. An animal study found that GSK2586881 administration effectively cleaves Ang II and restores normal BP in a mouse model with acute Ang II-dependent hypertension [39]. Furthermore, GSK2586881 alleviates cardiac remodeling induced by pressure overload and Ang II, and improves diabetic kidney disease in Akita mice by restoring normal BP and decreasing albuminuria [40]. Direct administration of GSK2586881 results in anti-remodeling in animal models of pulmonary hypertension, reduced pulmonary vascular remodeling in the murine bleomycin model [41], and reverses established pulmonary hypertension in a mouse model of familial pulmonary arterial hypertension [42]. In clinical trials, GSK2586881 rapidly reduces Ang II levels, often falling below the quantification threshold in healthy humans and in participants with pulmonary arterial hypertension [37, 38]. Studies in animal models and humans indicate that GSK2586881 is safe and well-tolerated, with potentially beneficial effects on BP, renal function, and pulmonary hypertension. No severe adverse events or evidence of immunogenicity were observed in participants with pulmonary arterial hypertension who received GSK2586881 at doses ranging from 0.1 to 0.8 mg/kg [38]. Compared to traditional anti-hypertensive medications, GSK2586881 demonstrates additional cardio-protective and reno-protective effects, while also being beneficial for pulmonary hypertension. However, further research is required to ascertain the detailed mechanisms for the hypotensive effects of GSK2586881.

### 5.2 Ang (1-7)

**Fig. 4.**
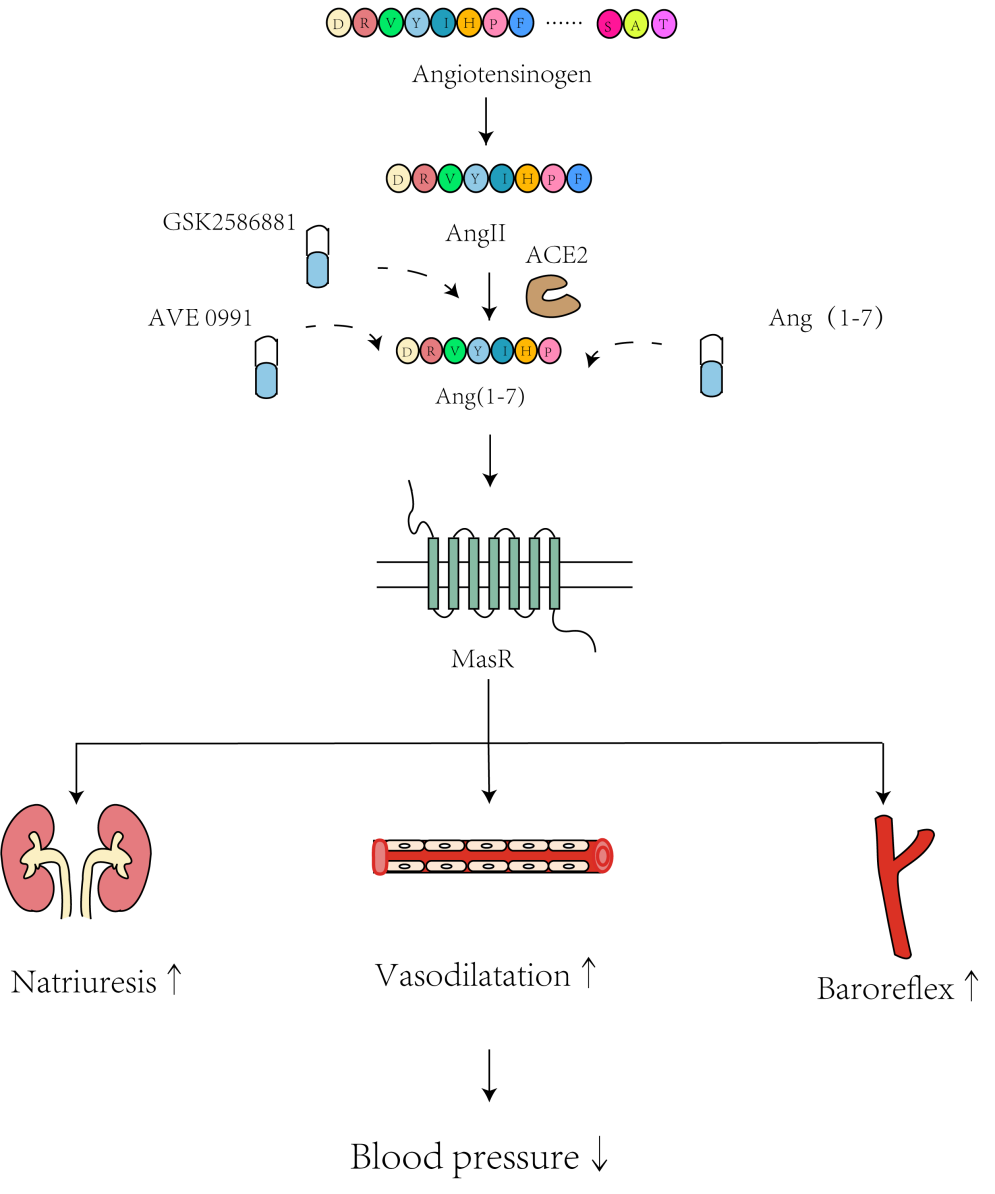
**Mechanisms underlying the hypotensive effects of GSK2586881, Ang (1-7), and AVE0991**. “Natriuresis↑” means the promotion of natriuresis; “vasodilatation↑” means the promotion of vasodilatation; “baroreflex↑” means the promotion of baroreflex; “blood pressure↓” means the decrease of blood pressure. Created with Adobe Illustrator 2023.

Several studies have consistently shown that administering Ang (1-7) has a protective effect on BP. Ang (1-7) in the peripheral circulatory system can impede the progression of chronic hypertension and the occurrence of end-organ damage in SHRs [43]. Administration of Ang (1-7) in preeclampsia (PE) mice significantly reduced the SBP induced by agonistic autoantibodies to the AT1R, which plays a crucial role in the development of PE [44]. Furthermore, Ang (1-7) was shown to reduce BP and promote relaxation of the mesenteric arteries in normotensive rat models [45]. In addition to hypertensive and normotensive models, oral Ang (1-7) treatment for 6 weeks in rats with metabolic syndrome was found to reduce MAP compared to high-fat diet-vehicle rats [46]. Importantly, pretreatment with Ang (1-7) was shown to suppress the Ang II-induced increase in BP and heart rate, the pressor reaction and vasoconstriction, as well as its impact on relaxation induced by acetylcholine in SHRs [47]. However, Ang (1-7) can have varying impacts on BP regulation depending on the location and method of administration. For example, injecting Ang (1-7) into the rostral ventrolateral medulla (RVLM) of renovascular hypertensive rats resulted in elevation of MAP and increased renal sympathetic nerve activity [48]. Additionally, following subcutaneous delivery of Ang (1-7) or iodo-Ang (1-7) through a 28-day osmotic mini pump, SHRs showed increased BP with negligible impact on heart rate and cognitive function [49]. An important drawback of administering Ang (1-7) externally is that its peptide composition leads to a short biological half-life, limited oral bioavailability, and reduced stability. Because of these limitations, Ang (1-7) is often delivered subcutaneously with osmotic mini pumps. Ang (1-7) may regulate BP through natriuresis, vasodilatation, and baroreflex activation (Fig. 4).

#### 5.2.1 Natriuresis

Early study suggested that administration of Ang (1-7) could lead to increased natriuresis, diuresis, and glomerular filtration rate [50]. Additionally, Ang (1-7) was observed to reduce the activity of sodium (Na^+^)-adenosine 5^′^-triphosphate (ATP)ase stimulated by Ang II in isolated proximal tubules, indicating a direct effect on sodium transport in the tubules [51]. Further laboratory studies found that Ang (1-7) acts as a vasodilator within the kidneys, enhancing the excretion of sodium and water by reducing the activity of Na^+^/hydrogen (H^+^) exchanger-3 (NHE-3) in the proximal tubules [52]. O’Neill *et al*. [53] recently showed that intrarenal MasR plays a role in the diuretic and natriuretic effects induced by Ang (1-7). The extent of these effects was dependent on the sodium level in the diet, since Ang (1-7) enhances the elimination of water and salt after consuming a low sodium diet, but reduces these effects with a high sodium diet. Therefore, the ability of Ang (1-7) to improve fluid excretion appears to be associated with the level of RAS activation [53].

#### 5.2.2 Vasodilatation

Recent research has demonstrated that Ang (1-7) exerts its effects in peripheral tissues through vasodilatation. Ang (1-7) was found to promote relaxation of the middle cerebral artery in dogs by triggering NO secretion from endothelial cells following activation of endothelial nitric oxide synthase (eNOS) [54]. Activation of MasR by Ang (1-7) can also improve endothelial dysfunction and reduce the harmful impact of Ang II on vascular tension and on the Ang II-induced pressor response by utilizing the NO-cyclic guanosine monophosphate (cGMP)-protein kinase G (PKG) pathway [55]. This highlights the crucial role of NO in facilitating the action of Ang (1-7) and its potential therapeutic consequences.

#### 5.2.3 The Activation of the Baroreflex

Physiologically, activation of the baroreflex leads to a reflexive reduction in BP. Facilitation of the baroreflex induced by Ang (1-7), observed after both peripheral and intra-cerebroventricular administration, has notable significance [56, 57]. Activation of the baroreflex is therefore a plausible mechanism that contributes to the alleviation of BP induced by Ang (1-7).

### 5.3 AVE0991

AVE0991 is a nonpeptide analog of the Ang (1-7) peptide that can be taken orally. It replicates the effects of Ang (1-7) in various organs, but with a longer half-life and increased stability compared to Ang (1-7) [58]. This analog protects against end-organ damage induced by N-nitro-L-arginine methylester (L-NAME) in hypertensive rats, as well as in SHRs [59]. AVE0991 enhances endothelial function in rats by stimulating MasR and promoting NO synthesis [60]. Furthermore, it controls the systolic BP and the rate of increase in maximal left ventricular pressure in rats with streptozotocin-induced diabetes [61]. Concurrent administration of small amounts of aliskiren and AVE0991 synergistically reduced the BP in rats with DOCA-induced hypertension, suggesting superior efficacy compared to individual administration of these medications [62]. It is worth noting that AVE0991 induces a hypotensive effect primarily in hypertensive situations, rather than in normotensive conditions. AVE0991 decreases BP in rats with two-kidney one-clip (2K1C) hypertension, enhancing both the slowdown and acceleration of the baroreflex, but has no effect on baseline BP in normotensive sham rats [63]. Another study reported there were no notable alterations in the BP of diabetic rodents [58]. The ability of AVE0991 to lower BP depends on the presence of undamaged endothelium. Nevertheless, the impairment of vascular endothelial function is widely acknowledged as a major factor in the secondary complications associated with diabetes [64], and hence this result may be due to the damaged endothelium in diabetic rats. In a recent comprehensive analysis, AVE0991 demonstrated encouraging anti-hypertensive properties, together with decreased inflammation, cardiac remodeling, fibrosis, and oxidative stress, thus highlighting its potential cardio-renal benefits [65].

In summary, Ang (1-7) and its analog AVE0991 are novel therapeutic targets in the RAS that exert anti-hypertensive effects through various mechanisms other than targeting the renin/ACE2/Ang (1-7)/MasR axis. AVE0991 may also have potential cardio-renal benefits compared with traditional anti-hypertensive medicine. However, a recent study found that mice treated with Ang (1-7) and AVE0991 exhibited more severe albuminuria compared to the PE group, suggesting potential renal risks [44]. Therefore, further research is warranted to evaluate the adverse events and pharmacokinetic characteristics of Ang (1-7) and AVE0991.

## 6. Focus on the Alamandine/MrgD Receptor Axis

The MrgD receptor is found in nociceptive neurons in muscles, the heart and testes. Functional studies in blood vessels and transfected cells indicate that it serves as the receptor for alamandine. Alamandine has been observed in arterial smooth muscle cells, endothelial cells positive for eNOS, and atherosclerotic plaques in the cardiovascular system [33, 66]. Ang (1-7) and alamandine share almost identical amino acid sequences, differing only in the presence of asparatic acid/alanine at the start of the chain, thus explaining their analogous physiological characteristics.

### Alamandine

Lautner *et al*. [33] showed that alamandine induces a hypotensive effect, as demonstrated by a persistent reduction in high BP when administered orally in SHRs. Furthermore, alamandine effectively lowered BP when injected into the caudal ventrolateral medulla of Fischer rats and 2K1C hypertensive rats [33, 67]. A recent study by Hekmat *et al*. [68] in 2K1C hypertensive rats found a notable decline in MAP, left-ventricular systolic pressure, SBP and DBP during a two-week infusion of alamandine, accompanied by an increase in the maximum rate of left ventricular pressure. Intraperitoneal administration of alamandine for 6 weeks significantly decreased SBP, DBP, and MAP compared to the control, in contrast to the effects observed in hydralazine-treated rats [69]. Moreover, Gong *et al*. [70] reported that alamandine reduced SBP, DBP, and MAP in Dahl rats fed high-salt diets.

**Fig. 5.**
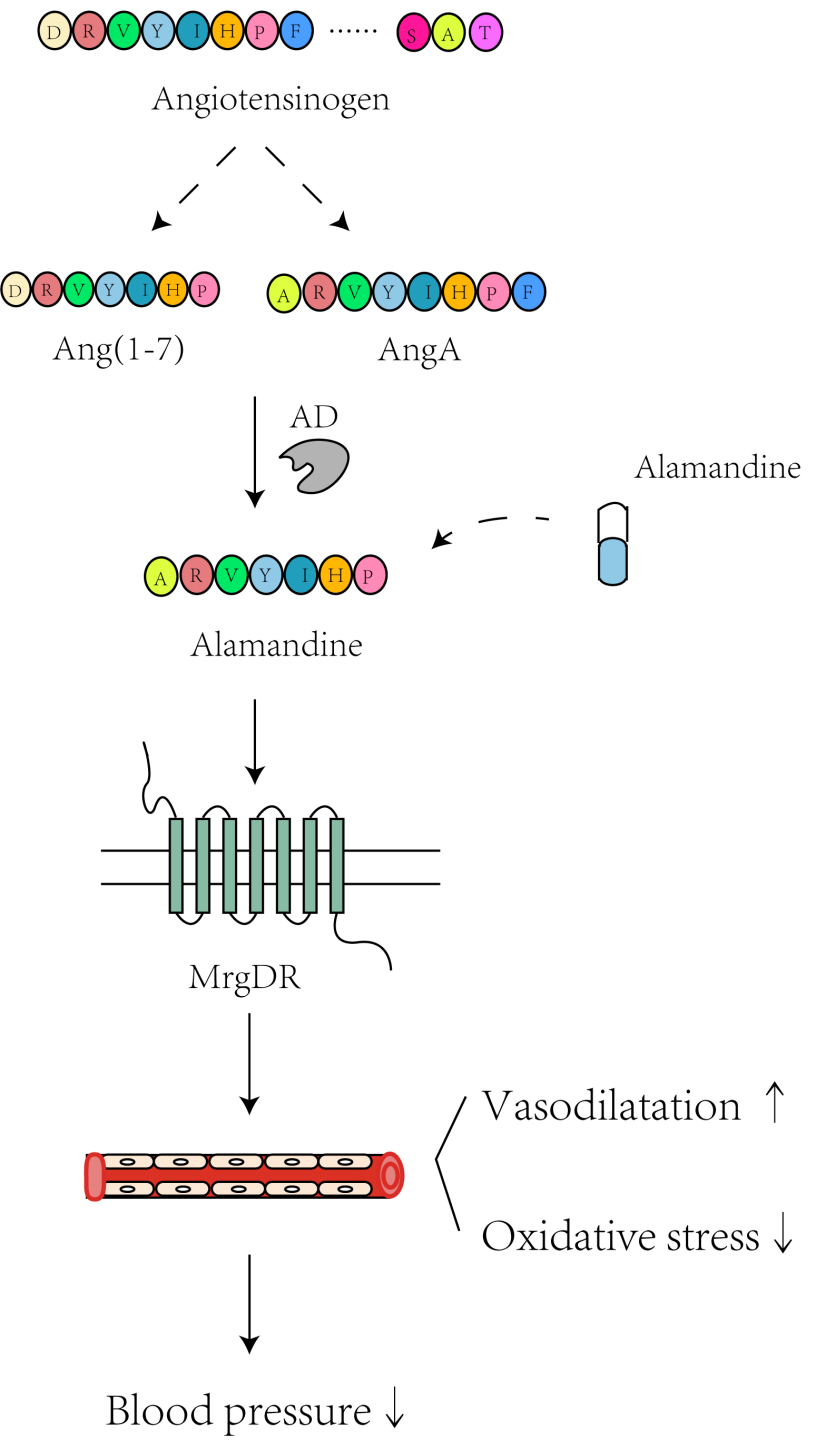
**The hypotensive mechanisms involving alamandine targeting of the alamandine/MrgD receptor (MrgDR) axis**. “Vasodilatation↑” means the promotion of vasodilatation; “oxidative stress↓” means the inhibition of oxidative stress; “blood pressure↓” means the decrease of blood pressure. Created with Adobe Illustrator 2023.

The proposed mechanisms underlying the hypotensive effect of alamandine are inhibition of oxidative stress and vasorelaxation mediated by NO release (Fig. 5). Alamandine is thought to be a crucial suppressor of oxidative stress, playing a critical role in the development of kidney dysfunction and hypertension [71]. Additionally, the alamandine-induced vasodilation of aortic rings and expansion of blood vessels can be reduced by the NO synthase inhibitor, L-NAME. Alamandine has been shown to enhance the activation of eNOS and subsequent NO production by activating the MrgD receptor, thereby exerting its vasodilatory and anti-hypertensive effects [33]. Parmentier *et al*. [72] found that increased vasorelaxation following alamandine treatment of stroke-prone SHRs was due to the release of NO and prostaglandins. Compared with existing anti-hypertensive therapies, alamandine could potentially offer better outcomes for heart enlargement, left ventricular function, and kidney dysfunction. Few clinical studies have so far evaluated the pharmacokinetic properties and side effects of alamandine, and further research on this topic is warranted.

## 7. Focus on the Renin/ACE/Ang II/AT2R Axis

Current evidence supports a counter-regulatory role for AT2R, whereby it counteracts the detrimental effects mediated by AT1R. It does this by promoting vasodilation, enhancing sodium excretion, improving baroreflex, exerting neuroprotective effects, and triggering anti-inflammatory, anti-proliferative, and anti-fibrotic responses [73]. These effects are achieved through the secretion of vasodilatory substances such as NO and bradykinin (BK) [74]. The AT2R signaling pathway involves cGMP and the activation of PKG and p38 mitogen-activated protein kinase (MAPK) [75]. AT2Rs are distributed in various body systems, including the cardiac system, blood vessels (especially on endothelial cells), central nervous system, and immune cells [76, 77].

### 7.1 C21

C21 is an orally accessible, selective, high-affinity AT2R agonist with a low molecular weight. It exhibits low affinity towards several other receptors, including AT1R and MasR, with a 4000-fold higher selectivity for AT2R than AT1R. Hence, C21 effectively activates AT2R without affecting AT1R, showcasing its specificity [78]. C21 has 20–30% oral bioavailability and an estimated half-life of 4 h in rats. Protective effects of C21 on the heart, brain, and kidneys have been reported, along with its anti-inflammatory properties [79]. C21 has demonstrated efficacy for improving BP in various animal models of hypertension. Notably, it showed effectiveness in pregnant hypertensive Sprague Dawley rats, non-pregnant female Sprague Dawley rats induced with Ang II, and male obese Zucker rats induced with salt. Additionally, C21 administration to dams exposed to perfluoro-octane sulfonic acid (PFOS), a reproductive toxicant, effectively prevented a rise in BP [80, 81, 82]. Preclinical experiments with C21 have also shown an ability to decrease pulmonary hypertension in rodent models caused by monocrotaline or pulmonary fibrosis [83]. Moreover, C21 can induce a hypotensive effect via different methods of administration, including intraventricular and intrarenal administration. A study in both normotensive rats and SHRs has demonstrated that continuous ICV injection administration of C21 results in decreased BP, an increase in spontaneous baroreflex sensitivity, reduced plasma norepinephrine levels, and the suppression of sympathetic outflow [74]. A hypotensive effect was also observed in awake normotensive Sprague-Dawley rats following ICV C21 treatment, as well as in rats with cardiac insufficiency [84, 85]. Additionally, ICV delivery of C21 has been shown to attenuate the increase in BP caused by DOCA/NaCl in female rats, thus providing direct evidence that central AT2R activation causes the hypotensive effect [86]. A recent study showed that local administration of C21 into the RVLM consistently lowered BP through AT2R activation. This effect was accompanied by an increase in the local level of gamma-aminobutyric acid (GABA) and a requirement for functional GABA receptors. A contradictory report indicated that immediate administration of C21 into the nucleus of the solitary tract (NTS) did not impact the GABA level in the same nucleus [87]. Another study found that direct administration of C21 into the paraventricular nucleus did not reduce the BP or alter the local concentration of GABA [88]. However, a recent investigation found that continuous use of C21 led to reduced mRNA levels of GABA-producing enzymes and a decrease in BP. These effects were only observed when AT2R was present on GABAergic neurons in the NTS [89]. The exact cause of the hypotensive effect induced by C21 in the central pivot is still uncertain. However, the hypotensive response to the central delivery of C21 is likely to be influenced, to some extent, by the activation of AT2R in the RVLM and NTS.

**Fig. 6.**
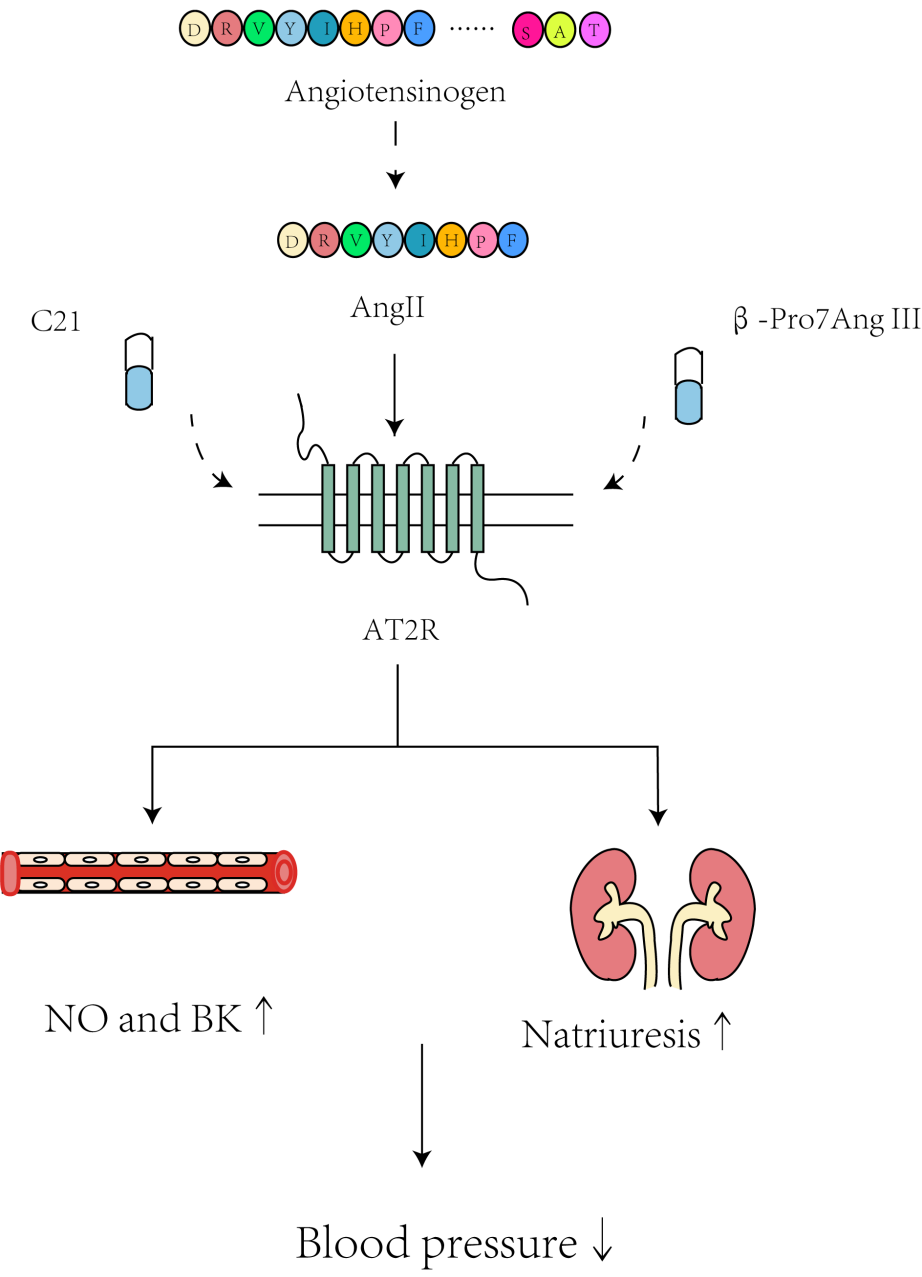
**Hypotensive mechanisms of C21 and β-Pro7Ang III by targeting the renin/ACE/Ang II/AT2R axis**. NO, nitric oxide; BK, bradykinin. “NO and BK↑” means the increase of NO and BK production; “natriuresis↑” means the promotion of natriuresis; “blood pressure↓” means the decrease of blood pressure. Created with Adobe Illustrator 2023.

Intrarenal infusion of C21 significantly attenuated the rise in BP caused by systemic Ang II infusion in Sprague-Dawley rats [90]. Additionally, chronic C21-induced AT2R activation initiated and sustained the translocation of AT2R to the apical plasma membrane (APM) of renal proximal tubular cells (RPTCs). Importantly, the systemic or intrarenal administration of C21 was found to be successful at lowering BP even without simultaneous inhibition of AT1R, confirming its autonomous ability to reduce hypertension [91]. However, a recent study in SHRs showed that despite intrarenal C21 infusion, MAP remained elevated but was still within the normotensive range [92]. This highlights the need to further investigate the specific mechanisms and conditions under which C21 exerts its anti-hypertensive effects. These may be related to NO, BK, and natriuresis, as shown in Fig. 6.

#### 7.1.1 NO and BK

Previous research indicates that C21 enhances NO-mediated relaxation in rats with hypertension and hyperandrogenism, suggesting that AT2R activation may increase the synthesis of NO by endothelial cells [80]. AT2R-null rats had significantly lower levels of BK and cGMP in Ang II-induced renal interstitial fluid compared to wild-type controls, indicating that BK, NO, and cGMP are involved in this response [93]. Additionally, C21 reversed the decline in endothelial eNOS protein levels in the vessels and uterine arteries of C21-treated hypertensive pregnant dams, while also increasing plasma BK levels, suggesting that AT2R activation could enhance endothelial function [82]. The hypothesis that C21 directly facilitates eNOS expression is supported by previous evidence that C21 increases eNOS expression in cultured cardiomyocytes through the calcineurin/nuclear factors of activated T cells (NF-AT) pathway [94]. AT2R activation also resulted in increased synthesis of BK, which can then enhance vasodilation by triggering the synthesis of NO, prostacyclin, and endothelium-derived hyperpolarizing factors [80].

#### 7.1.2 Natriuresis

Various experimental models have consistently demonstrated the natriuretic effect of AT2R. In the 2K1C rat model, C21 infusion was observed to simultaneously enhance urine Na^+^ excretion (UNaV), fractional excretion of Na^+^ (FENa), and fractional excretion of lithium (FELi) [90]. Similarly, Ali and Hussain [81] showed that intravenous infusion of C21 in obese Zucker rats at a 16-fold higher dose (5.0 µg/[kg⋅min]) resulted in elevated levels of UNaV, FENa, and FELi. These findings indicate the diuretic effect of AT2Rs occurs primarily in the proximal tubule of the kidney via activation of the BK/NO/cGMP signaling cascade [95]. Moreover, the activation of AT2R, whether acutely or with long-term use of C21, causes AT2R to relocate to the APMs of RPTCs. This mechanism is believed to enhance and sustain the natriuretic response. Additionally, the main transporters responsible for moving sodium across the basolateral membrane of RPTCs, NHE-3 and Na^+^/potassium (K^+^)-ATPase, are internalized within the cell in a BK/NO/cGMP-dependent manner [82, 90]. Consequently, the localization of AT2Rs on the upper plasma membrane is likely to act as a facilitating mechanism for AT2Rs in the tubule, hindering the reabsorption of sodium. Together, these results highlight the multifaceted role of AT2Rs in regulating renal sodium.

### 7.2 β-Pro7Ang III 

A promising alternative to C21 as a novel AT2R agonist is the development of β-Pro7-Ang III. This compound exhibits remarkable AT2R selectivity, with a >20,000-fold selectivity over AT1R. *In vitro* studies have demonstrated the AT2R agonist properties of β-Pro7-Ang III, highlighting its ability to induce vasorelaxation. Moreover, experiments using conscious SHRs and diabetic-hypertensive rats have shown that β-Pro7-Ang III acutely lowers BP, particularly during low-level ARB [96, 97]. Recent studies have shed light on the long-term benefits of β-Pro7-Ang III, and in particular its anti-fibrotic and anti-inflammatory properties. These effects were observed in mice fed a high-salt diet, and in rats with diabetes and hypertension. The findings suggest that β-Pro7-Ang III holds promise as a therapeutic intervention for cardiovascular and metabolic conditions. However, further research is needed to characterize the detailed mechanisms underlying its hypotensive effects, and to evaluate the adverse events and pharmacokinetic characteristics associated with its clinical use.

In summary, C21 and β-Pro7-Ang III are novel AT2R agonists with high selectivity for AT2R, making them effective anti-hypertensive agents with few impacts on AT1R. Moreover, β-Pro7-Ang III appears to have additional anti-fibrotic and anti-inflammatory properties compared to traditional anti-hypertensive medications.

## 8. Focus on Brain Ang III

Brain Ang III is formed from brain Ang II by APA and has been identified as a key active peptide in the brain RAS. Through interaction with AT1R, brain Ang III amplifies the release of systemic vasopressin and has a consistent stimulating effect on BP in conscious SHRs and DOCA-salt rats. Notably, both of these rat models show elevated activity of the brain RAS [98]. Targeting of APA, the enzyme responsible for generating brain Ang III, is therefore a promising strategy for managing hypertension based on the central nervous system.

### 8.1 EC33 and Firibastat

Administration of the APA inhibitor EC33 by ICV injection significantly lowers BP in alert SHRs, whereas intravenous administration has no impact, even at high doses. These findings suggest that EC33-induced BP reduction is due to the inhibition of brain Ang III production rather than to systemic effects [99]. However, following oral administration, EC33 has to cross the digestive system, liver and BBB. The prodrug firibastat was developed to overcome this limitation. It consists of two EC33 molecules connected by a disulfide bridge, and can cross the BBB when given systemically [100]. In humans, a single oral dose of firibastat is absorbed via the gastrointestinal tract with a median of 1.5 h (range 0.75–3 h) after administration. Following oral administration in hypertensive DOCA-salt rats or SHRs, firibastat crosses the intestinal, hepatic, and blood-brain barriers and enters the brain [101]. Once in the brain, cerebral reductases cleave the disulfide bridge to produce two active EC33 molecules. These molecules inhibit brain APA activity in hypertensive rats, thus preventing brain Ang III formation and consequently lowering BP [102]. The BP starts to decrease 2 h after oral firibastat administration, is maximal between 5 and 9 h after administration, and persists for up to 15 h. The effect of the drug is no longer detectable 24 h after administration [101]. Conscious SHRs show reduced brain APA activity and a dose-dependent decrease in BP after oral firibastat administration, while systemic RAS activity remains unaffected [103]. A significant reduction in SBP was observed in hypertensive DOCA-salt rats 24 days after firibastat administration, with no development of tolerance noted [100]. In SHRs, co-administration of oral firibastat with the systemic RAS inhibitor enalapril enhanced the BP-lowering effect, suggesting a collaborative impact for the suppression of systemic and cerebral RAS functions. However, in DOCA-salt rats, the combination of firibastat and enalapril resulted in minimal reduction of BP, indicating a lack of synergy between the inhibition of brain APA and blocking systemic ACE activity in this hypertensive rat model. The pharmacological mechanisms responsible for the effect remain unclear. It is also worth noting that repeated oral administration of firibastat did not induce hypotensive effects in normotensive rats and normotensive Beagle dogs [100, 103], suggesting a lack of response in normotensive animals. Healthy volunteers showed good tolerance to firibastat in the NCT01900171 Phase I trial, with single oral doses of up to 2000 mg, and twice-daily doses of up to 750 mg. Importantly, these doses did not impact BP, pulse rate, or overall RAS function [104]. In a pilot Phase IIa trial, 34 individuals with mild-to-moderate hypertension were administered firibastat at a dose of 1000 mg/d for 4 weeks (250 mg twice daily for the first week, followed by an increase to 500 mg twice daily for the next three weeks). This resulted in a reduction of 2.7 mmHg in daytime systolic ambulatory blood pressure, and 4.7 mmHg in systolic office BP compared to the placebo. No alterations in biochemical safety parameters were noted [105]. During the NEW-HOPE trial (Phase IIb, NCT03198793), firibastat treatment was associated with adverse events in 14.1% of the 107 subjects. Additionally, 7.5% of subjects discontinued therapy due to adverse events, with headache (4.3%) and skin responses (3.1%) being the most prevalent adverse events. Of the five reported serious adverse events, only one was linked to firibastat administration. The occurrence of skin lesions attributed to firibastat may be explained by the sulfhydryl content in EC33, as previous reports have documented eruptions caused by sulfhydryl drugs [106, 107]. Importantly, no notable changes in clinical laboratory results were noted throughout the trial [108]. *In vitro* experiments showed no significant inhibition of human recombinant cytochrome P450s (CYP) (CYP1A2, CYP2C9, CYP2C19, CYP2D6, and CYP3A4) by firibastat or EC33 at a concentration of 10 µmol/L [104]. Together, these studies demonstrate the favorable tolerability and safety profile of firibastat, supporting its clinical use in patients.

**Fig. 7.**
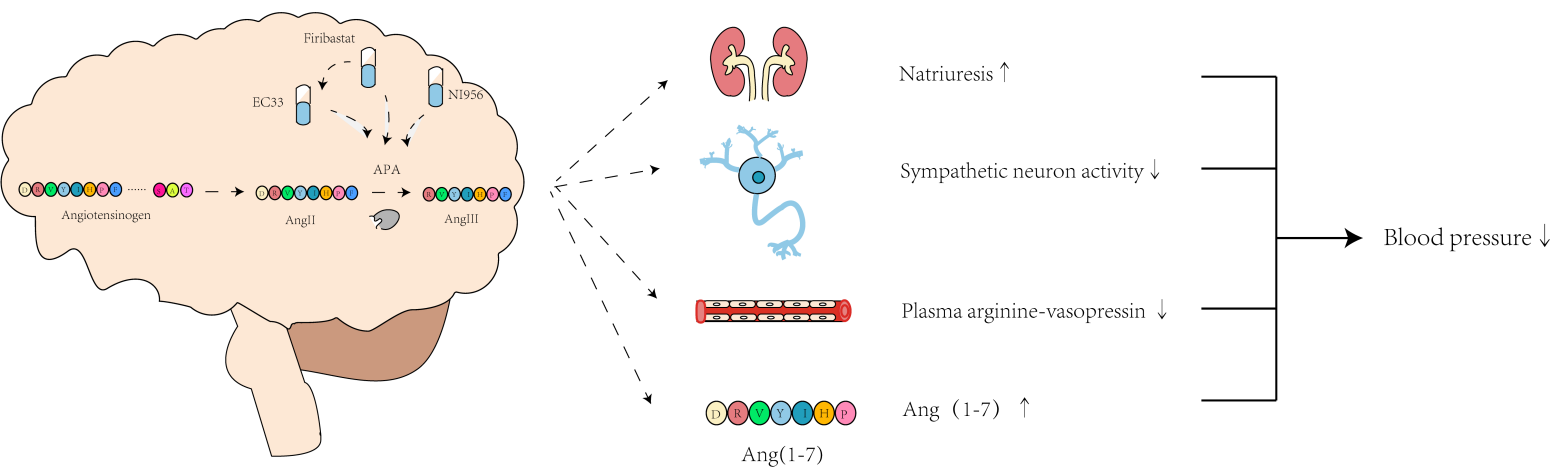
**Hypotensive mechanisms of NI956, EC33, and firibastat in targeting the brain RAS**. “Natriuresis↑” means the promotion of natriuresis; “sympathetic neuron activity↓” means the decrease of sympathetic neuron activity; “plasma arginine-vasopressin↓” means the reduction of plasma arginine-vasopressin concentration; “Ang (1-7)↑” means the increase of Ang (1-7) production; “blood pressure↓” means the decrease of blood pressure. Created with Adobe Illustrator 2023.

Firibastat and EC33 can induce a hypotensive effect through multiple mechanisms (Fig. 7). Initially, they reduce plasma arginine-vasopressin levels and enhance diuresis, thus contributing to lower BP by reducing the blood volume [102]. Additionally, these drugs inhibit sympathetic neuron activity, thereby decreasing vascular resistance and improving baroreflex function [103]. Furthermore, firibastat has been hypothesized to hinder the activation of brain AT1R by Ang III, thus reducing Ang III formation in the brain. This in turn increases the conversion of brain Ang II into Ang (1-7), which subsequently activates MasR and reduces BP. These diverse mechanisms highlight the potential of firibastat as a novel medication for hypertension treatment through its influence on the brain RAS.

### 8.2 NI956

Keck *et al*. [109] recently described a new prodrug for APA inhibition. NI956 was developed through the disulfide bridge-mediated dimerization of NI929. NI956 is 10-fold more potent than EC33 at inhibiting recombinant mouse APA activity *in vitro* [110]. Oral administration of NI956 (4 mg/kg) to hypertensive DOCA-salt rats resulted in a significant decrease in MAP within 3 to 10 h after treatment, with an effective dose of 0.53 mg/kg. Similar to prior investigations with firibastat, the MAP of normotensive Wistar-Kyoto (WKY) rats did not change following oral NI956 intake. The contrasting impact of NI956 on brain APA function and arterial BP in normotensive WKY and hypertensive DOCA-salt rats can be attributed to the elevated activity of brain APA and RAS in DOCA-salt rats, but its absence in normotensive WKY rats. Additionally, NI956 was found to decrease plasma arginine-vasopressin levels and enhance diuresis and natriuresis, potentially contributing to a reduction in BP without affecting plasma sodium and potassium levels [109].

In summary, EC33, firibastat and NI956 are novel anti-hypertensive medications that target brain APA. EC33 and firibastat exhibit favorable tolerability and safety profiles. They do not significantly inhibit human recombinant cytochrome P450s, indicating they have only a slight impact on liver injury and on drug interactions metabolized by the liver. NI956 is 10-fold more potent than EC33 at inhibiting recombinant mouse APA activity, without affecting plasma sodium and potassium levels, thus highlighting its potentially strong anti-hypertensive effects and safety profile.

## 9. Conclusions

Following discovery of the RAS in 1898, a growing number of studies have established its crucial role in blood regulation. Investigation of the RAS has led to the identification of several potential therapeutic targets, including the renin/(pro)renin/PRR axis, renin/ACE/Ang II/AT1R axis, etc. To date, however, only two anti-hypertensive medications have been approved by the FDA: AT1R blockers and direct renin inhibitors. Moreover, almost 20 years have passed since the last anti-hypertensive agent targeting the RAS was approved by the FDA (aliskiren). This review has summarized the latest research and mechanisms involving novel anti-hypertensive medications that target the RAS and which may be introduced for the future treatment of hypertensive patients. Although these new agents have shown effectiveness in some studies, several limitations remain to be addressed. Firstly, the optimal route of drug administration requires confirmation. For example, alamandine and C21 can induce an anti-hypertensive effect via different modes of administration, and finding the optimal route of delivery requires further study. Secondly, few pharmacokinetic studies have been carried out on drugs such as AVE0991, PRO20, Ang (1-7), alamandine, β-Pro7-Ang III and NI956. More research is therefore needed to assess their anti-hypertensive effects and pharmacokinetic characteristics using different hypertensive models. Thirdly, some novel medications that target the RAS, such as zilebesiran and firibastat, are still undergoing clinical trials. Their anti-hypertensive and adverse effects require further evaluation in larger groups of hypertensive patients, as well as their interactions with other drugs.

Emerging evidence has shown that sodium-glucose cotransporter 2 inhibitors (SGLT2i) may be beneficial for cardiovascular diseases such as hypertension and heart failure [111]. Whether SGLT2i can exert anti-hypertensive effects by acting on RAS is still unknown, and further research on the mechanism of these inhibitors is warranted. In line with therapeutic advances in other areas such as oncology, the application of molecular therapies may become a new focus of anti-hypertensive treatment in the future. Classical anti-hypertensive medications can cause adverse effects due to their low selectivity. For example, spironolactone has major adverse effects such as painful gynecomastia and impotence due to its non-selective binding to androgen and progesterone receptors. The discovery of siRNA was recognized by the award of the 2006 Nobel Prize for Physiology or Medicine. This novel therapeutic strategy enables selective reduction in the expression of specific proteins, leading to the development of a growing number of novel drugs. For example, inclisiran significantly reduces circulating levels of low-density lipoprotein cholesterol (LDL-C) by inhibiting the expression of proprotein convertase subtilisin/kexin type 9 (PCSK9). In addition to their high selectivity, siRNAs display good safety and tolerability profiles. Zilebesiran is the first anti-hypertensive medication based on siRNA and targets the production of hepatic AGT. It has shown a potent hypotensive effect, high efficiency, and good safety profile with a persistent anti-hypertensive effect lasting for 6 months in current clinical trials. KARDIA-2 is a randomized, double-blind, placebo-controlled, multicenter clinical trial designed to compare the efficacy and safety of zilebesiran when combined with the classical anti-hypertensives. The completion of this trial is expected in 2025, with approval by the FDA expected to follow several years later. Other novel anti-hypertensive medications based on siRNA are likely to be developed in the near future.
